# Spontaneous tauopathy with parkinsonism in an aged cynomolgus macaque

**DOI:** 10.3389/fnagi.2026.1715911

**Published:** 2026-01-28

**Authors:** Mirai Takahashi, Daisuke Taniguchi, Manabu Funayama, Ettore Cioffi, Shusei Fukuyama, Takuya Ito, Toshi Nakajima, Ko Tsuyama, Yuanzhe Li, Satoshi Ishiko, Satomi Chiken, Asuka Nakajima, Taku Hatano, Yoshikazu Tasaki, Kazuko Hasegawa, Yasushi Shimo, Atsushi Nambu, Nobutaka Hattori, Kaoru Takakusaki

**Affiliations:** 1 Department of Physiology, Division of Neuroscience, Faculty of Medicine, Asahikawa Medical University, Asahikawa, Hokkaido, Japan; 2 Department of Neurosurgery, Faculty of Medicine, Asahikawa Medical University, Asahikawa, Hokkaido, Japan; 3 Department of Neurology, Faculty of Medicine, Juntendo University, Tokyo, Japan; 4 Research Institute for Diseases of Old Age, Graduate School of Medicine, Juntendo University, Tokyo, Japan; 5 International Collaborative Research Administration, Juntendo University, Tokyo, Japan; 6 Department of Medico-Surgical Sciences and Biotechnologies, University of Rome Sapienza, Latina, Italy; 7 Innovation Management Organization, Chiba University, Chiba, Japan; 8 Department of Physiology, Faculty of Medicine, Kindai University, Osaka, Japan; 9 Department of Ophthalmology, Faculty of Medicine, Asahikawa Medical University, Asahikawa, Hokkaido, Japan; 10 National Institute for Physiological Sciences, Okazaki, Aichi, Japan; 11 Department of Neurology, Juntendo University Nerima Hospital, Tokyo, Japan; 12 Department of Hospital Pharmacy and Pharmacology, Asahikawa Medical University, Asahikawa, Hokkaido, Japan; 13 Division of Neurology, NHO Sagamihara National Hospital, Sagamihara, Kanagawa, Japan; 14 Neurodegenerative Disorders Collaboration Laboratory, RIKEN Center for Brain Science, Saitama, Japan

**Keywords:** corticobasal degeneration, cynomolgus macaques, parkinsonism, progressive supranuclear palsy, tauopathy

## Abstract

**Introduction:**

Aged non-human primates have been reported to develop tau pathology; however, most studies lack evidence of any associated neurological symptoms. To determine whether spontaneous tauopathy in cynomolgus macaques manifests with neurological symptoms, we evaluated a symptomatic aged monkey (Monkey T) alongside an asymptomatic control (Monkey A).

**Methods:**

Two male cynomolgus macaques, aged 33–34 years old at the time of necropsy, were examined. They were evaluated using comprehensive behavioral, pathological, and genetic analyses.

**Results:**

Monkey T exhibited progressive neurological symptoms for approximately two years prior to euthanasia, including tremors, nuchal dystonia, and a flexed posture, whereas Monkey A showed no abnormalities. Monkey T demonstrated persistent tremors (6.9 ± 0.7 Hz) and reduced daily motor activity, with modest improvement following L-DOPA administration. Neuropathological evaluation revealed brainstem atrophy and mild depigmentation of the substantia nigra and locus coeruleus. Extensive phosphorylated tau accumulation was observed throughout the brainstem tegmentum, including neurofibrillary tangles, threads, coiled bodies, and astrocytic inclusions. All tau lesions were positive for 4-repeat tau and negative for 3-repeat tau. MAPT sequencing identified four non-pathogenic 3′UTR variants differing between the two monkeys. Isoform analysis showed balanced 3R/4R tau expression in Monkey A but an approximately 1.3-fold increase in 4R tau in Monkey T.

**Discussion:**

The parkinsonian symptoms observed in Monkey T were more likely attributable to widespread tau pathology in the brainstem rather than overt degeneration of the nigrostriatal dopaminergic system. This case represents a rare instance of spontaneous tauopathy in an aged cynomolgus macaque, a condition that is extremely difficult to reproduce experimentally. These findings highlight the potential value of cynomolgus macaques as a relevant model for studying sporadic tauopathies, including tau seeding mechanisms.

## 1 Introduction

Tauopathies are neurodegenerative diseases that are characterized by the accumulation of misfolded tau protein in neurons and glial cells; they include conditions such as Alzheimer’s disease (AD), progressive supranuclear palsy (PSP), and corticobasal degeneration (CBD) (Olfati et al., 2022). To elucidate their underlying mechanisms, various rodent models have been developed using genetic modifications or prion-like propagation techniques (Duyckaerts et al., 2008; Iba et al., 2013; King et al., 2021; Yanamandra et al., 2013). However, these models have inherent limitations; for example, they have differences in brain size and structural complexity compared with humans, and the models have a limited capacity to replicate the predominantly sporadic forms of human tauopathies (Chen and Zhang, 2022; Olfati et al., 2022).

Non-human primates, particularly cynomolgus macaques, are phylogenetically close to humans and possess relatively complex brain architecture. They are capable of performing tasks that assess human-like cognitive and motor functions, and they express all six isoforms of tau with over 98% sequence homology to the longest human isoform (Qamar and Visanji, 2023). These anatomical, functional, and molecular similarities make cynomolgus macaques a valuable model for investigating the pathophysiology of human tauopathies. A recent study demonstrated that tau seeds derived from human brains with PSP can induce both the pathological and motor features of PSP when injected into cynomolgus macaques (Darricau et al., 2023). Although exogenous tau administration is a powerful approach for modeling disease mechanisms, it remains essential to determine whether naturally occurring tau pathology can give rise to parkinsonian symptoms, to establish macaques as a relevant model for sporadic tauopathies.

Previous studies have reported that aged cynomolgus macaques exhibit tau accumulation in addition to amyloid-*β* deposition (Beckman et al., 2024; Iwaide et al., 2023; Paspalas et al., 2018), with regional patterns resembling either AD or 4-repeat tauopathies such as PSP and CBD (Oikawa et al., 2010; Uchihara et al., 2016). In both reports, however, neurological symptoms were not described, and behavioral assessments were not performed. To our knowledge, only one prior case of tauopathy in a cynomolgus macaque presenting with parkinsonian symptoms has been reported (Kiatipattanasakul et al., 2000). This animal exhibited tremor, gait disturbance, and reduced activity, and neuropathological examination revealed neurofibrillary tangles as well as glial tau inclusions. It therefore remains unclear whether tau pathology alone is sufficient to cause neurological symptoms in cynomolgus macaques.

In the current study, we observed a cynomolgus macaque with pathologically confirmed tauopathy who exhibited neurological symptoms including tremors, reduced activity, nuchal dystonia, and a flexed posture. Unlike the previously reported case, we conducted detailed behavioral assessments using continuous video monitoring and evaluated the effects of L-DOPA administration, thereby allowing for a more comprehensive characterization of the clinical features. We also observed an asymptomatic control monkey housed in the same captive environment for the same duration. This case provides additional evidence that non-transgenic, non-seeded cynomolgus macaques can spontaneously develop symptomatic tauopathy, thus reinforcing their potential utility as a model for human tauopathies.

## 2 Materials and methods

### 2.1 Subjects

#### 2.1.1 Monkey T (symptomatic animal)

We refer to the symptomatic cynomolgus macaque with tauopathy and parkinsonism, including tremor, as Monkey T (symptomatic animal with tauopathy). Monkey T was a male adult cynomolgus macaque (*Macaca fascicularis*) that had been introduced into the animal laboratory at Asahikawa Medical University more than three decades previously by a commercial laboratory animal vendor. Upon arrival, Monkey T weighed 2.0 kg and was estimated to be 2–3 years old (age at sacrifice: 33–34 years). He was maintained for long-term observation as part of an aging-related study (Animal experiment approval number: R4-027-03). Throughout the study period, the animal was housed individually in a metal cage (70 cm × 70 cm × 84 cm) under controlled conditions: a constant temperature of 25 °C, a 14-h light/10-h dark cycle (lights on at 6:00 a.m. and off at 8:00 p.m.), and standard laboratory chow and water were available ad libitum. No experimental or invasive procedures were performed during the observation period.

Over the course of observation, Monkey T began to exhibit neurological symptoms. The animal had shown both resting and active tremors involving all four limbs for approximately 2 years before the study, and decreased food intake was observed from 9 months prior to the experiment. These signs progressed gradually, and in the months preceding the study, the animal adopted a flexed posture, exhibited nuchal dystonia, and showed reduced voluntary movement. The final body weight of Monkey T was 5.0 kg.

#### 2.1.2 Monkey A (asymptomatic aged control animal)

Monkey A was another male adult cynomolgus macaque that was housed in the same laboratory under identical environmental conditions and for the same duration as Monkey T. His body weight at arrival was 2.0 kg, and his estimated age was 2–3 years (age at sacrifice: 33–34 years). He was also part of the aging observation study, as was Monkey T, but did not show any abnormal behaviors or neurological symptoms throughout the observation period. The final body weight of Monkey A was 6.5 kg.

### 2.2 L-DOPA administration

**Figure 1:**
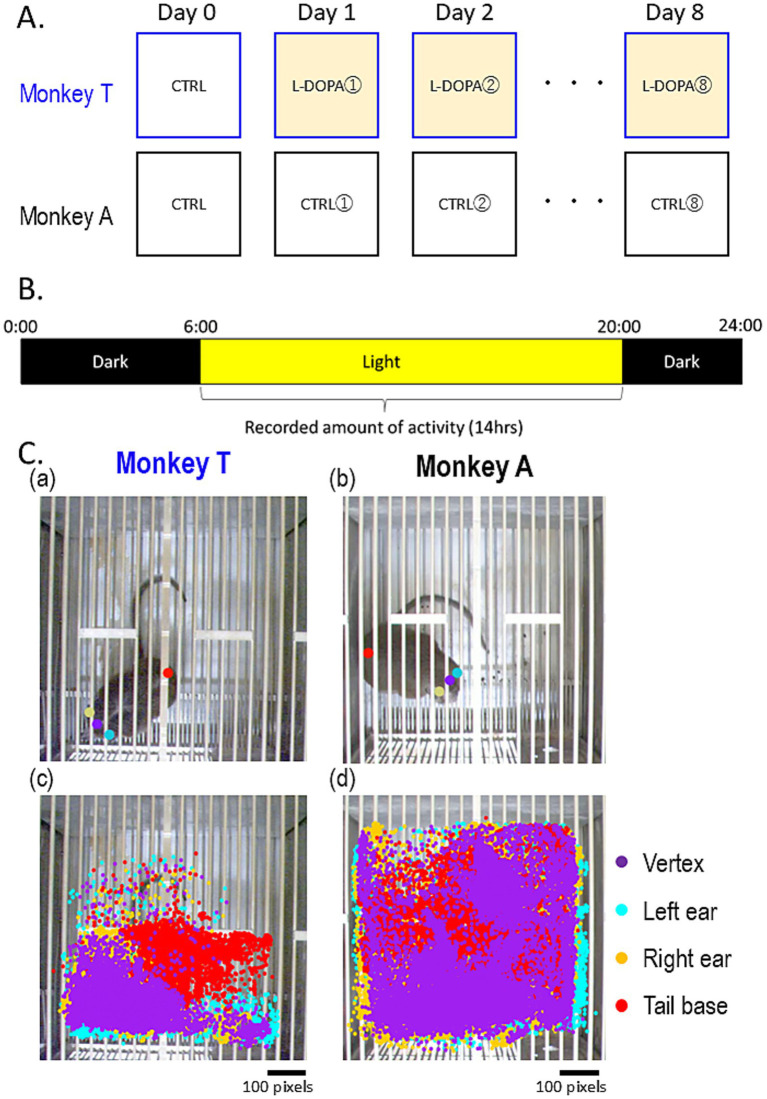
Behavioral analysis. **(A)** Schedules of control (CTRL) and L-DOPA administration. **(B)** The light/dark cycle in the monkeys’ breeding room. **(C)** Estimated body part positions using DeepLabCut software. **(a,b)** Representative estimated body parts of Monkeys T **(a)** and A **(b)**. **(c,d)** Range of body part distribution of Monkeys T **(c)** and A **(d)**. Scatter plots indicating body part distribution on the control day. Each dot indicates the position of each body part at any given second during the control day.

To evaluate the effects of dopaminergic treatment, Monkey T received oral administration of levodopa carbidopa hydrate (L-DOPA, 200 mg/day; NEODOPASTON COMBINATION TABLETS L100, Ohara Pharmaceutical Co., Ltd.) for 8 consecutive days. The medication was mixed with food and administered twice daily. The dosing schedule is illustrated in Figure 1A.

### 2.3 Behavioral analysis

To quantify daily activity levels, video recording of the monkeys’ behavior was conducted. A ceiling-mounted camera system (VR-24, TEAC Co., Ltd.) recorded images at a rate of 1 frame/s during the 14-h light period (Figure 1B). Video data were analyzed offline using DeepLabCut (version 2.2.3), a markerless pose estimation software (Mathis et al., 2018). The program was trained to estimate the positions of key anatomical landmarks, including the vertex, left and right ears, and tail base.

For training, 40 frames from 2 days of video for Monkey T and 180 frames from 7 days of video for Monkey A were manually labeled. Ninety-five percent of the labeled frames were used for training. A ResNet-50-based neural network with default parameters was trained for 1 million iterations and refined through 21 and 16 separate training sessions for Monkey T and Monkey A, respectively. All labeled outputs were manually reviewed for accuracy. Daily activity was quantified by calculating the total movement distance of the tracked body parts. The Mann–Whitney U test was used to compare movement speeds between the two monkeys.

Monkey T exhibited tremors. To analyze tremor frequency, additional high-frame-rate video was recorded at 30 frames/s (FDR-AX45, Sony Corp.). Tremor frequency was calculated by averaging the number of oscillations/s over a 24-s interval.

### 2.4 Pathological analysis

#### 2.4.1 Euthanasia and tissue collection

Following completion of the original experimental protocol, both monkeys were euthanized and perfused under the supervision of a veterinarian. All procedures were performed in accordance with the Guidelines for the Care and Use of Nonhuman Primates in Neuroscience Research, issued by the Japan Neuroscience Society, and were approved by the Animal Studies Committee of Asahikawa Medical University. Under sedation and analgesia with intramuscular ketamine hydrochloride (25 mg/kg), blood was collected from the femoral vein, and cerebrospinal fluid was obtained via the foramen magnum for future analyses. Deep anesthesia was then induced using intraperitoneal pentobarbital sodium (50 mg/kg), followed by transcardial perfusion with 4 L of 0.01 M phosphate-buffered saline over a 15-min period using a roller pump. Upon completion of perfusion, the entire brain was harvested, along with other major organs, for subsequent analysis.

#### 2.4.2 Immunohistochemistry

The left hemisphere was immersion-fixed in 10% paraformaldehyde, and selected brain regions were embedded in paraffin. Paraffin-embedded tissue was sectioned at 6 μm thickness using a microtome, and sections were stained with hematoxylin and eosin and Gallyas–Braak silver staining for histological evaluation. The right hemisphere was sliced and frozen for later biochemical assays. For immunohistochemistry, antigen retrieval was performed by autoclaving sections in citrate buffer at 110 °C for 10 min. After treatment with blocking solution, sections were incubated overnight at 4 °C with the following primary antibodies: anti-phosphorylated alpha-synuclein (pSyn#64, monoclonal, 1:1,000; Wako), anti-phosphorylated tau (AT8, monoclonal, 1:100; Thermo Fisher Scientific), anti-4-repeat tau (RD4, monoclonal, 1:100; Merck Millipore), anti-3-repeat tau (RD3, monoclonal, 1:100; Merck Millipore), anti-amyloid-*β* (Aβ_1–42_, polyclonal, 1:100; IBL), and anti-tyrosine hydroxylase (TH; monoclonal, 1:1000; Sigma-Aldrich). Antibody binding was visualized using a peroxidase-polymer-based detection system (Histofine Simple Stain MAX-PO kit; Nichirei) with diaminobenzidine as the chromogen. For confocal microscopy, sections were similarly processed for antigen retrieval before being incubated in blocking solution (Cat# 06349–64, Nacalai Tesque) for 10 min. This was followed by overnight incubation at 4 °C with primary antibodies, and then a 1-h incubation at room temperature with Alexa Fluor Plus 488, 594, or 647-conjugated secondary antibodies (Thermo Fisher Scientific). Autofluorescence was quenched using a lipofuscin quencher (Cat# 23014, Biotium) and tissue was mounted with a 4′,6-diamidino-2-phenylindole-containing medium (Cat# H-1800, Vector Laboratories). Confocal images were acquired using a TCS SP5 microscope (Leica). Digital images were processed and analyzed using ImageJ software (version 1.53b).^1^

#### 2.4.3 Quantification of TH-positive neurons

The number of TH-positive neurons in the substantia nigra was determined from high-magnification images of TH-immunostained sections. For each monkey, a coronal midbrain section at the level containing the oculomotor nerve was selected. The substantia nigra was manually delineated as the region of interest based on anatomical landmarks. TH-positive neurons were manually counted using the Cell Counter plugin in Fiji/ImageJ (version 1.53b). Counts were performed independently by three blinded raters, and the results are presented as the mean ± standard deviation of their measurements.

#### 2.4.4 Western blot analysis

Sarkosyl-insoluble fractions were prepared from the pons of both monkey and human tauopathy brain tissue, as previously described (Taniguchi et al., 2024). For the monkey samples, equal amounts of tissue (100 mg) were dissected from the same anatomical region (pons) of each animal and processed in parallel using an identical protocol. For immunoblotting, the insoluble pellets were resuspended in phosphate-buffered saline via sonication, mixed with sodium dodecyl sulfate sample buffer, and boiled for 5 min. Proteins were then separated on a 10–20% gradient polyacrylamide gel and transferred to membranes for immunodetection. Sarkosyl-insoluble tau was detected using the following primary antibodies: TauC (1:4000) for full-length tau, RD3 (1:1000) for 3-repeat tau, and anti-4-repeat tau (1:1000, Cosmo Bio) for 4-repeat tau. Phosphorylated tau was detected using AT8. Because conventional housekeeping proteins and total protein staining did not provide a stable reference in the sarkosyl-insoluble fraction, immunoblot data were interpreted based on strictly matched tissue input and parallel sample processing. The use of human tauopathy brain tissue samples in this study was approved by the Ethics Committee of the Juntendo University School of Medicine (approval number: M19–0235).

### 2.5 Genetic analysis

#### 2.5.1 Sequencing of the *Macaca fascicularis* microtubule-associated protein tau (*MAPT*) gene

According to the Ensembl database,^2^ *M. fascicularis* possesses 21 chromosomes and 22,504 genes. The *MAPT* gene is located on chromosome 16 of the *M. fascicularis* genome (Cunningham et al., 2022). Although the NCBI Gene database lists 10 *MAPT* isoforms, the University of California, Santa Cruz (UCSC) Genome Browser registers 18 (Kent et al., 2002). This discrepancy reflects differences in database versions and annotation strategies. For the present study, we used XM_005584536.1—available only in the UCSC database—as the reference sequence because it includes 15 exons and has the most comprehensive isoform coverage. Although it is labeled as a predicted transcript in the NCBI database, our comparison confirmed that it encompasses all known isoforms. Polymerase chain reaction (PCR) primers were designed using Primer3web and targeted all exons, splice junctions, and untranslated regions (UTRs) of the reference sequence (Untergasser et al., 2012). The primer sequences are provided in Supplementary Table 1. Genomic DNA was extracted from the peripheral blood of Monkeys T and A using the QIAamp DNA Blood Maxi Kit (Qiagen, Venlo, Netherlands). PCR amplification was performed using AmpliTaq Gold 360 DNA Polymerase (Thermo Fisher Scientific). The PCR products were purified with ExoSAP-IT (Thermo Fisher Scientific) and sequenced via the Sanger method using the primers listed in Supplementary Table 1. To identify potential variants, the resulting sequences were aligned to the reference sequence using the UCSC BLAT tool (Kent, 2002). Based on previous report, primers were designed to distinguish 3-repeat (3R) and 4-repeat (4R) tau in *M. fascicularis* MAPT (NCBI Gene ID: 102119414) by the molecular weight of PCR products, depending on the presence or absence of exon 10 (McMillan et al., 2008). The primer sequences were as follows: forward (exon 9), 5′-TCCACTGAGAACCTGAAG-3′; and reverse (exon 11), 5′-TCCTGGTTTATGATGGATGTT-3′. Total RNA was extracted from fresh-frozen brains of Monkeys using RNeasy Plus Mini Kit (Qiagen, Venlo, Netherlands). cDNA was synthesized from total RNA using ReverTra Ace qPCR RT Master Mix (TOYOBO, Osaka, Japan). After endpoint PCR, amplicons were electrophoresed using the MultiNA microchip electrophoresis system (Shimadzu, Kyoto, Japan). The molar concentrations of 3R and 4R tau PCR products were quantified based on the molar concentration of the molecular weight marker and band intensity.

### 2.6 Statistical analysis

The Mann–Whitney U test was used to compare movement speed between the two monkeys. For group comparisons of pathological assessments, Student’s t-test was applied. A *p*-value < 0.05 was considered significant.

## 3 Results

### 3.1 Daily activity

**Figure 2:**
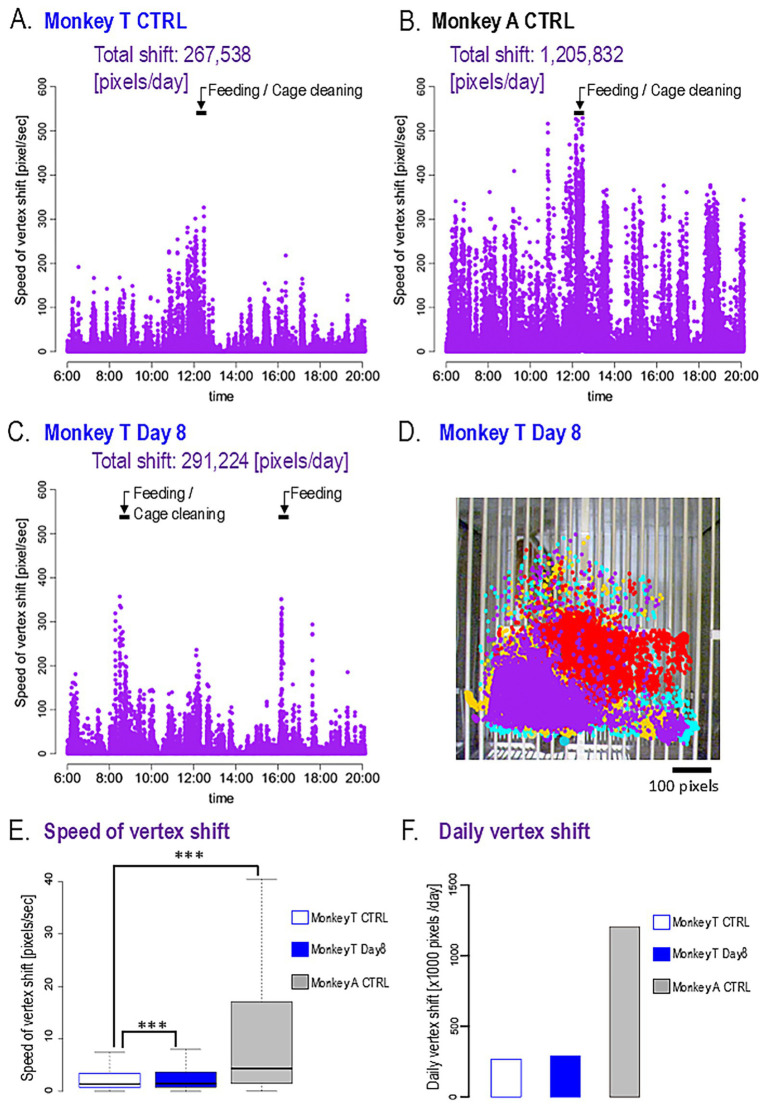
Daily activities. **(A,B)** The speed of vertex movement of Monkeys T **(A)** and A **(B)** during the control (CTRL) day. The timing of animal care is indicated by arrows. The total number of vertex shifts is displayed at the top. **(C,D)** Effects of L-DOPA on the daily activities of Monkey T. **(C)** Speed of vertex movement and **(D)** distribution of vertex position. **(E)** Box and whisker plot of vertex shift speed. ***p < 0.001. **(F)** Bar plot of daily vertex shift.

The range of movement across body parts is illustrated in a scatter plot (Figure 1C). Compared with Monkey A, Monkey T displayed a very restricted movement range. Furthermore, the mean speed of vertex movement was significantly slower in Monkey T than in Monkey A (1.39 vs. 4.34 pixels/s, *p* < 0.001; Figures 2A,B,E). Similarly, the total daily movement distance was substantially shorter in Monkey T than in Monkey A, although statistical analysis could not be performed due to the limited sample size (267,538 vs. 1,205,832 pixels/day, Figure 2F).

#### 3.1.1 Abnormal behaviors

Video-based behavioral analysis revealed that Monkey T exhibited tremors in all four limbs, occurring both at rest and during movement (Supplementary Videos 1, 2), with a mean frequency of 6.9 ± 0.7 Hz. Additional motor abnormalities included a pronounced flexed posture, nuchal dystonia, and dorsiflexed ankles while in a sitting position. When approached by an experimenter, the animal attempted to assume a threatening stance but was unable to stand (Supplementary Video 3). By contrast, Monkey A did not exhibit neurological symptoms (Supplementary Videos 4, 5).

### 3.2 Effects of oral L-DOPA in monkey T

Following 8 days of oral L-DOPA administration, Monkey T exhibited modest improvements in activity (Figure 2C). The movement range was slightly expanded (Figure 2D). The median vertex movement speed was significantly increased compared with baseline (CTRL vs. Day8; 1.39 vs. 1.47 pixels/s, *p* < 0.001, Figure 2E). Similarly, the total movement distance appeared to be increased, although statistical analysis could not be performed due to the limited sample size (CTRL vs. Day8; 267,538 vs. 291,224 pixels/day, Figure 2F). However, both metrics remained below those of Monkey A. Notably, Monkey T regained the ability to stand while displaying threatening behavior, although tremor persisted (Supplementary Video 6).

### 3.3 Neuropathological findings

#### 3.3.1 Macroscopic findings

**Figure 3:**
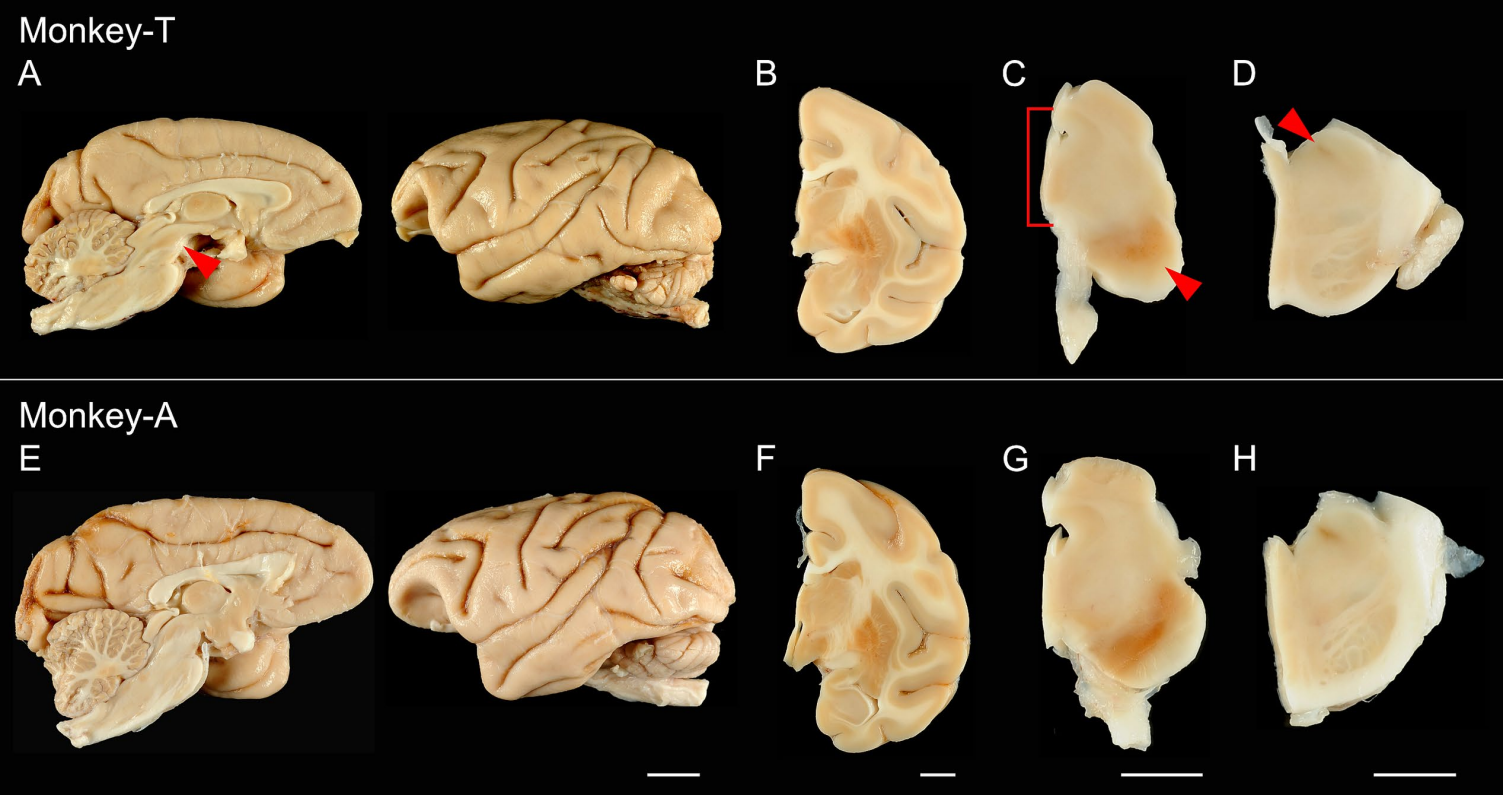
Macroscopic images of the brains from the two monkeys. Upper panels show images from Monkey T, and lower panels show the corresponding images from Monkey A. **(A,E)** Lateral and medial views of the hemispheres. Midbrain atrophy was suggested in Monkey T (red arrowhead). **(B,F)** Coronal sections at the level of the basal ganglia. **(C,G)** Axial sections of the midbrain. In Monkey T, the substantia nigra appeared depigmented (red arrowhead), and the anteroposterior diameter of the tegmentum was reduced (red bracket). **(D,H)** Axial sections of the pons. The locus coeruleus appeared depigmented in Monkey T (red arrowhead). Scale bar: 1 cm.

The brains of Monkeys T and A weighed 63 g and 71 g, respectively. While no definite cortical atrophy was observed laterally in either monkey, midbrain atrophy was suggested in Monkey T relative to Monkey A on the medial surface (Figures 3A,E, red arrowhead). The shape of the lenticular nucleus in the basal ganglia appeared comparable between the two monkeys (Figures 3B,F). In contrast, the brainstem of Monkey T appeared atrophic (Figures 3C,D), compared with Monkey A (Figures 3G,H). In Monkey T, the substantia nigra appeared depigmented (Figure 3C, red arrowhead), and the anteroposterior diameter of the tegmentum was reduced (Figure 3C, red bracket). At the level of the pons, the locus coeruleus also appeared depigmented in Monkey T (Figure 3D, red arrowhead).

#### 3.3.2 Microscopic findings

##### 3.3.2.1 Monkey T

**Figure 4:**
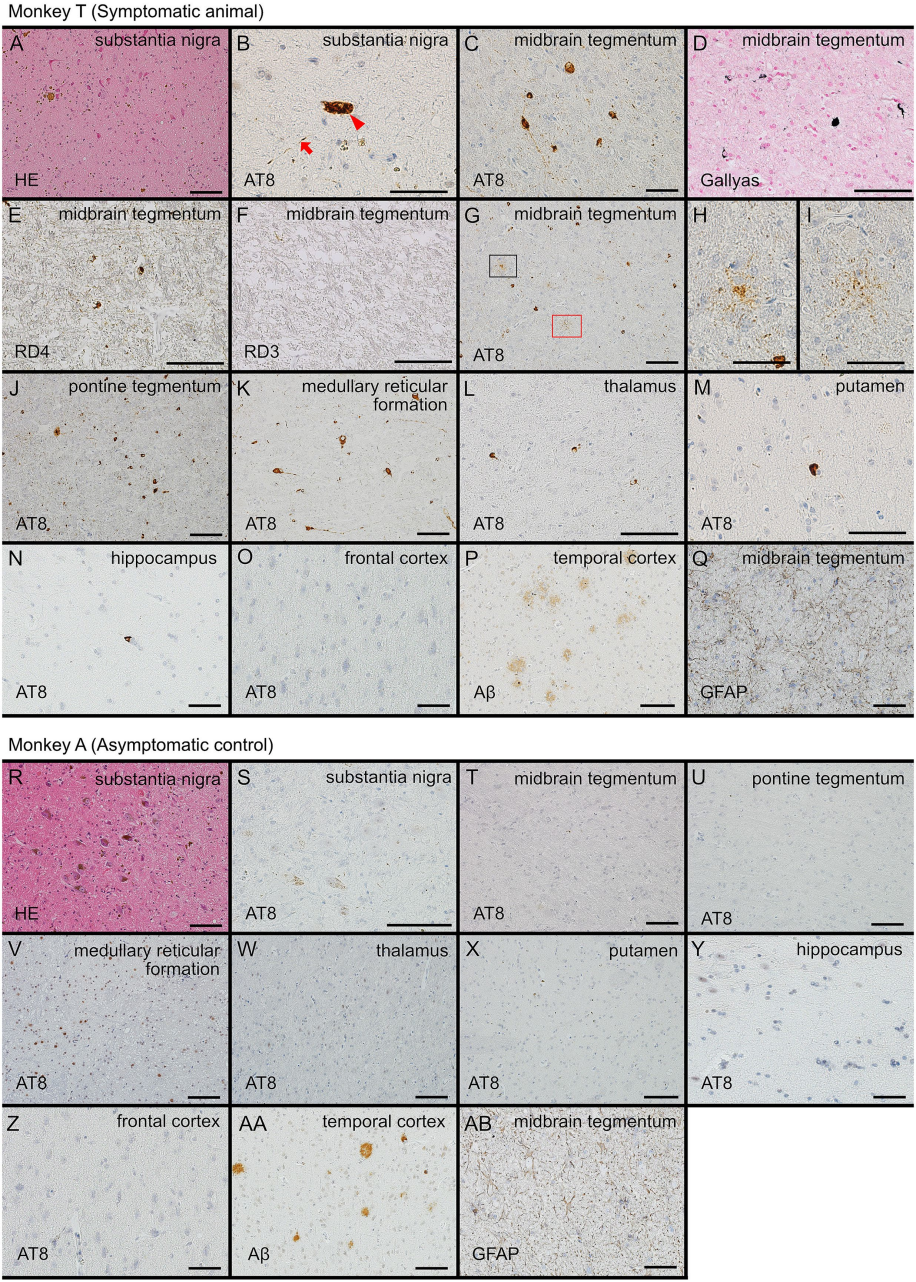
Microscopic images of the brains from the two monkeys. Panels **(A–Q)** show findings from Monkey T, and panels **(R–Y)** show findings from Monkey A. The anatomical regions and staining methods are indicated within each panel. **(A,R)** Hematoxylin and eosin staining of the substantia nigra, showing fewer pigmented neurons in Monkey T than in Monkey A. **(B)** Neurofibrillary tangles (red arrowhead) and thread-like tau inclusions (red arrow) in the substantia nigra. **(C–F)** Neurofibrillary tangles and threads in the midbrain tegmentum of Monkey T, detected by phospho-tau immunohistochemistry **(C)**, Gallyas–Braak silver staining **(D)**, and immunostaining for 4-repeat tau **(E)** but not 3-repeat tau **(F)**. **(G–I)** Low- and high-power views of astrocytic tau inclusions in the brainstem tegmentum. **(J–L)** Tau pathology enriched in the pontine tegmentum **(J)**, medullary reticular formation **(K)**, and extending into the thalamus **(L)**. **(M)** A small number of neurofibrillary tangles and threads in the putamen. **(N)** In the limbic system, only a few neurofibrillary tangles were detected in the hippocampus. **(O)** Tau pathology was absent in the cerebral cortex, including the frontal cortex of Monkey T. **(S–W)** In contrast, large-section immunohistochemical evaluation confirming the absence of tau pathology in the substantia nigra **(S)**, midbrain tegmentum **(T)**, pontine tegmentum **(U)**, medullary reticular formation **(V)**, thalamus **(W)**, putamen **(X)**, hippocampus **(Y)**, and frontal cortex **(Z)** of Monkey A. Occasional non-specific nuclear labeling with AT8 is observed in the medullary reticular formation **(V)**. **(P,AA)** A few diffuse amyloid plaques were identified in the cerebral cortex of both monkeys. **(Q,AB)** Neither monkey exhibited prominent astrogliosis, and no appreciable difference in gliosis was observed between Monkey T and Monkey A. Scale bar: 100 μm.

A reduction in pigmented neurons in the substantia nigra was evident in Monkey T on hematoxylin–eosin staining (Figure 4A). Immunohistochemistry for phosphorylated tau demonstrated neurofibrillary tangles (red arrowhead, Figure 4B) and thread-like inclusions (red arrow, Figure 4B) in this region. Neurofibrillary tangles and coiled bodies were more prominent in the midbrain tegmentum than in the substantia nigra (Figure 4C), and these inclusions were positive with Gallyas–Braak silver staining (Figure 4D). Immunolabeling demonstrated positivity for 4-repeat tau (Figure 4E) but negativity for 3-repeat tau (Figure 4F). Tau pathology was abundant in the brainstem tegmentum (Figure 4G), where astrocytic tau inclusions were also identified (Figures 4H,I). Tau pathology was enriched in the pontine tegmentum (Figure 4J) and medullary reticular formation (Figure 4K), and extended throughout the brainstem into the thalamus (Figure 4L). A small number of neurofibrillary tangles and thread-like inclusions were also observed in the putamen (Figure 4M). In the limbic system, only a few neurofibrillary tangles were detected in the hippocampus (Figure 4N), while tau pathology was absent in the remaining regions, including the cerebral cortex (Figure 4O).

##### 3.3.2.2 Monkey A

In contrast, Monkey A showed preservation of pigmented neurons in the substantia nigra (Figure 4R). Large-section immunohistochemical evaluation confirmed the absence of tau pathology in the substantia nigra (Figure 4S), midbrain tegmentum (Figure 4T), pontine tegmentum (Figure 4U), medullary reticular formation (Figure 4V), thalamus (Figure 4W), putamen (Figure 4X), hippocampus (Figure 4Y), frontal cortex (Figure 4Z), and other cortical regions.

#### 3.3.3 Co-pathology and glial response

In both monkeys, a few diffuse amyloid plaques were identified in the cerebral cortex (Figures 4P,AA). No phosphorylated *α*-synuclein–positive or TDP-43–positive pathology was detected in any brain region in either monkey. Regarding glial response, neither monkey exhibited prominent astrogliosis, and no appreciable difference in gliosis was observed between Monkey T and Monkey A (Figures 4Q,AB).

#### 3.3.4 Confocal imaging

**Figure 5:**
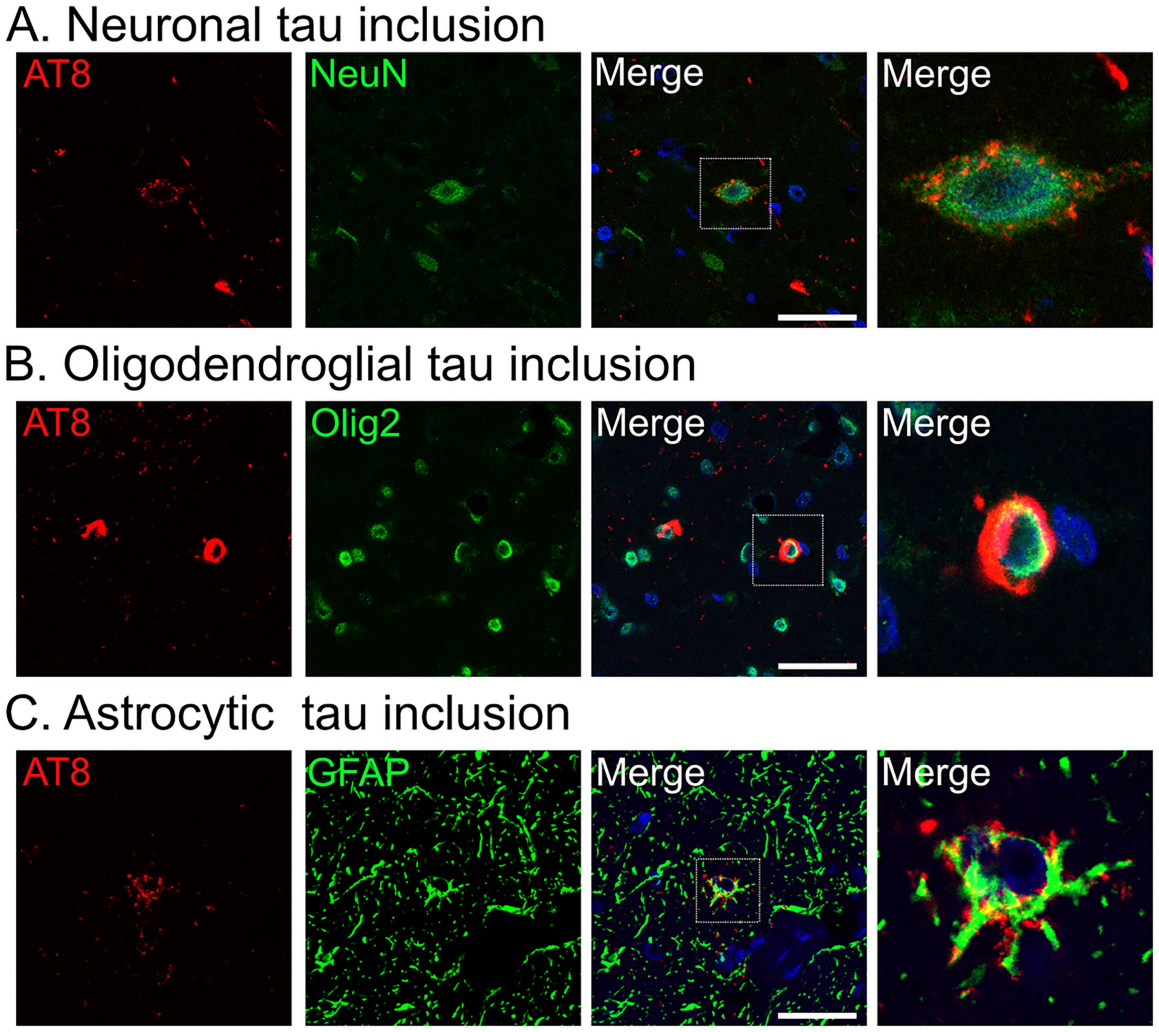
Confocal images of tau aggregates in the midbrain tegmentum of Monkey T. **(A–C)** Immunofluorescence labeling for phosphorylated tau (AT8, red) with neuronal (neuronal nuclei [NeuN]) **(A)**, oligodendroglial (oligodendrocyte transcription factor 2 [Olig2]) **(B)**, or astrocytic (glial fibrillary acidic protein [GFAP]) **(C)** markers (all in green), and nuclear counterstain with 4′,6-diamidino-2-phenylindole (blue). Scale bar: 10 μm.

Confocal imaging revealed that tau aggregates accumulated in neurons (Figure 5A) and oligodendrocytes (Figure 5B). In addition, a subset of tau inclusions observed in DAB-stained sections (Figures 4G–I) was confirmed to be localized within astrocytes, as demonstrated by co-localization of AT8 and GFAP (Figure 5C).

### 3.4 Nigrostriatal dopaminergic system

**Figure 6:**
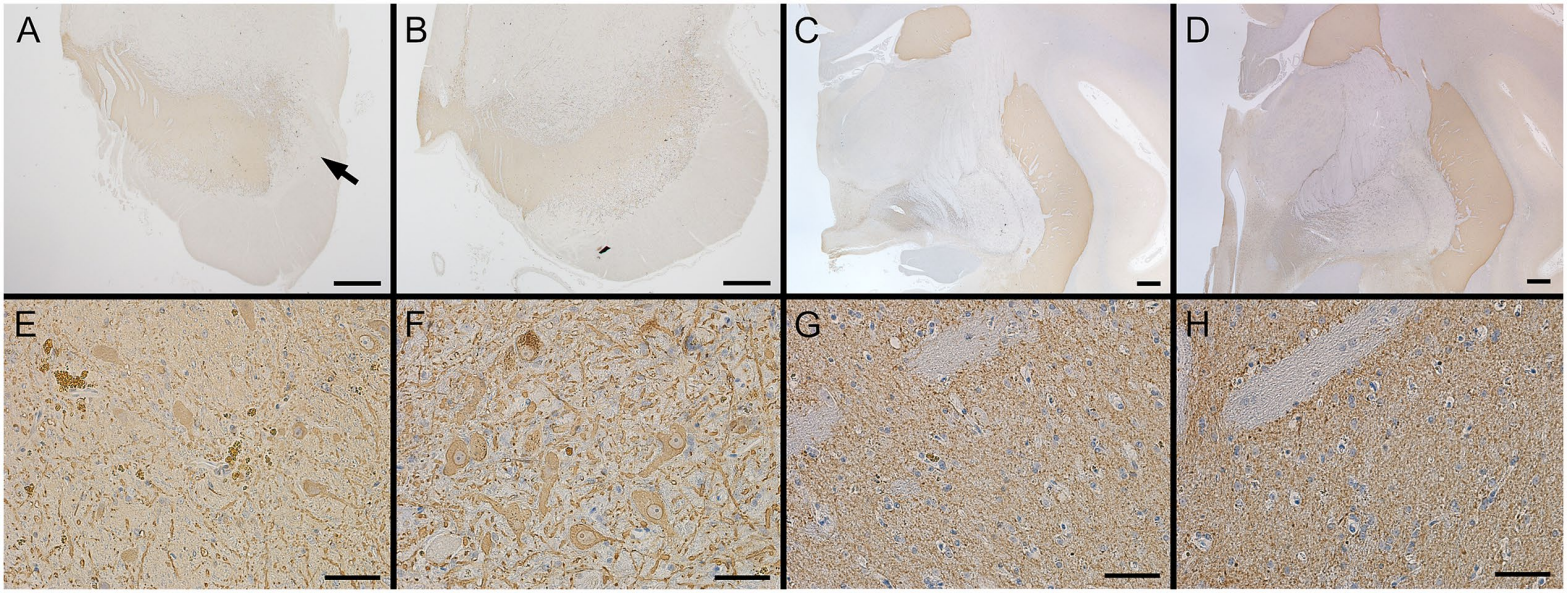
Tyrosine hydroxylase (TH) immunohistochemistry in the substantia nigra and striatum of the two monkeys. **(A–D)** Low-magnification views of the substantia nigra **(A,B)** and striatum **(C,D)** in Monkey T **(A,C)** and Monkey A **(B,D)**. The arrow in A indicates reduced TH immunoreactivity. **(E–H)** High-magnification views of the substantia nigra **(E,F)** and putamen **(G,H)** in Monkey T **(E,G)** and Monkey A **(F,H)**. Scale bars: 1 cm **(A–D)**, 50 μm **(E–H)**.

Macroscopic TH immunostaining in the substantia nigra revealed reduced lateral staining in Monkey T (Figure 6A, arrow) compared with the findings in Monkey A (Figure 6B). At the microscopic level, the number of TH-positive neurons in the substantia nigra was lower in Monkey T (307.3 ± 11.7) than in Monkey A (342.7 ± 18.6), representing an approximately 10% reduction (mean ± standard deviation from counts by three blinded raters; Figures 6E,F). By contrast, macroscopic examination of the striatum showed similar TH staining between Monkeys T (Figure 6C) and A (Figure 6D), consistent with the similar microscopic findings (Figures 6G,H).

### 3.5 Biochemical analysis of tau

**Figure 7:**
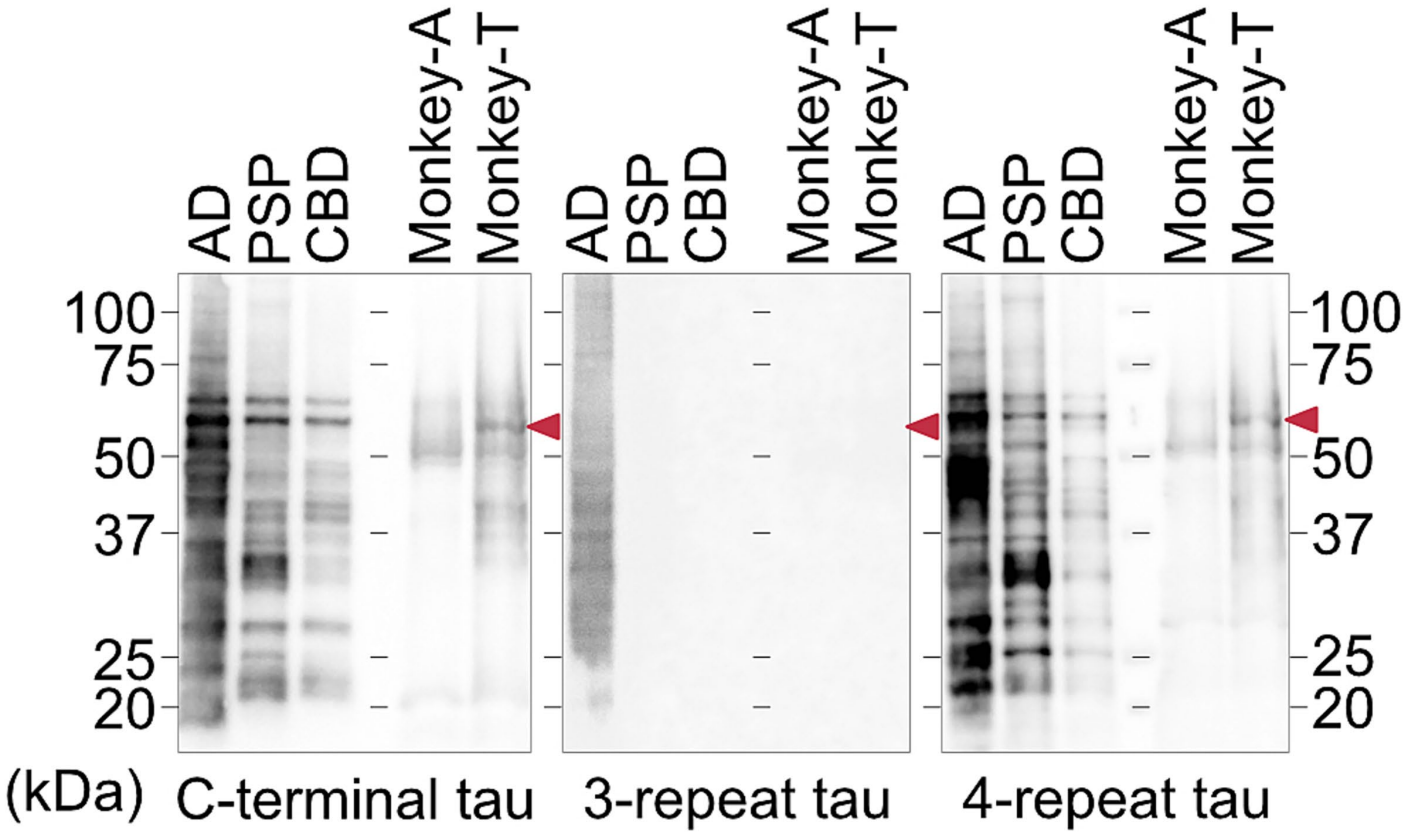
Biochemical characterization of tau fibrils in the two monkeys. Western blot analysis of sarkosyl-insoluble tau fractions prepared from the pons of Monkey T and Monkey A; brain samples from patients with Alzheimer’s disease (AD), progressive supranuclear palsy (PSP), and corticobasal degeneration (CBD) were included as disease controls. Full-length tau species are indicated by red arrowheads. Probed antibodies were total tau, 3-repeat tau, and 4-repeat tau.

Western blot analysis of sarkosyl-insoluble tau fractions was performed, with brain samples from patients with AD, PSP, and CBD included as disease controls (Figure 7). Consistent with the immunohistochemical findings, Monkey T exhibited a higher level of total tau than Monkey A. Both monkeys were negative for 3-repeat tau, whereas 4-repeat tau was more abundant in Monkey T than in Monkey A. In addition, immunoblot analysis using the AT8 antibody demonstrated stronger phosphorylated tau signals in Monkey T than in Monkey A (Supplementary Figure 1).

### 3.6 Sequence comparison of *MAPT* between Monkeys T and A

**Figure 8:**
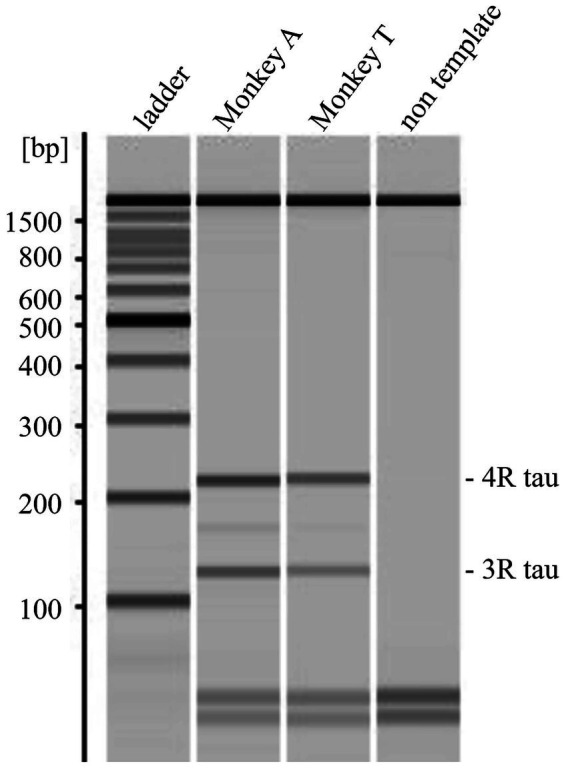
Comparison of tau isoform expression levels in two monkeys. Gel image showing detection of 3R-tau and 4R-tau isoforms by RT-PCR. Two PCR products were detected: 3R-tau at 126 bp and 4R-tau at 219 bp, differing in molecular weight due to the presence or absence of exon 10.

Sequence analysis revealed 12 single nucleotide variants (SNVs) in Monkeys T and A (Table 1). Of these, four SNVs exhibited different sequences between Monkeys T and A. Heterozygous g.58386636 C>T was detected in Monkey A only, heterozygous g.58386882 G>A was detected in Monkey A and homozygous g.58386882 G>A was detected in Monkey T, and heterozygous g.58387536 C>A and g.58388163G>C were detected in Monkey T only (Table 1, shown in bold). All four SNVs that differed in sequence between Monkeys T and A were located in the 3’-UTR region of *MAPT*. No variants with amino acid substitutions and no variants that would be expected to severely damage tau protein function were detected. A BLASTn-based homology analysis between *M. fascicularis* and *Homo sapiens* revealed a maximum sequence homology of 95%. No difference in homology was observed between the coding sequence and the UTRs. The four nucleotide differences between the two monkeys were conserved in human *MAPT* mRNA. To investigate whether these SNVs affect tau transcripts, we examined tau isoforms extracted from frozen monkey brains. RT-PCR products for 3R and 4R tau showed an approximate size difference of 93 bp, corresponding to the expected length of exon 10 (Figure 8). The molar concentration ratio (4R/3R) of about 126 bp 3R tau product to about 219 bp 4R tau product was 1.004 in Monkey A and 1.272 in Monkey T. Monkey A showed nearly equivalent expression of 3R and 4R tau, whereas Monkey T exhibited approximately 1.3-fold higher expression of the 4R isoform.

**Table 1:** Detected *MAPT* variants in the two monkeys.

*MAPT* exon	Monkey A	Monkey T
1	g.58257983 C>G (homo)	g.58257983 C>G (homo)
2	No SNVs	No SNVs
3	No SNVs	No SNVs
4	No SNVs	No SNVs
5	No SNVs	No SNVs
6	No SNVs	No SNVs
7	g.58348901C>G (het)	g.58348901C>G (het)
8	No SNVs	No SNVs
9	g.58352992 G>T (het), g.58353136 T>C (het)	g.58352992 G>T (het), g.58353136 T>C (het)
10	g.58355720 G>T (het), g.58355815 T>C (het)	g.58355720 G>T (het), g.58355815 T>C (het)
11	No SNVs	No SNVs
12	No SNVs	No SNVs
13	No SNVs	No SNVs
14	No SNVs	No SNVs
15	**g.58386636 C>T (het)**, **g.58386882 G>A (het)**, g.58388233 T>C (het), g.58388518 G>T (homo)	**g.58386882 G>A (homo)**, **g.58387536 C>A (het)**, **g.58388163G>C (het)**, g.58388233 T**>**C (het), g.58388518 G**>**T (homo)

het, heterozygous; homo, homozygous; SNVs, single nucleotide variants. Bold text denotes sequences that differ between Monkeys T and A.

## 4 Discussion

Here, we have presented a case of a cynomolgus macaque (Monkey T) that spontaneously developed symptomatic tauopathy. To characterize this case, we performed detailed pathological and behavioral analyses, including video-based monitoring. Findings were compared with those from a control monkey (Monkey A) that shared the same breeding history and environmental conditions. The neurological symptoms exhibited by Monkey T were atypical for human Parkinson’s disease. Tremors occurred not only at rest but also during posture maintenance, and had a frequency of 6.9 Hz, which is slightly higher than that typically observed in human Parkinson’s disease (Dirkx and Bologna, 2022). In addition, Monkey T displayed nuchal dystonia, a pronounced flexed posture, and markedly reduced daily motor activity compared with Monkey A. These symptoms exhibited only limited responsiveness to L-DOPA administration, suggesting a form of atypical parkinsonism (Postuma et al., 2015). In the present case, parkinsonian symptoms began to emerge around the age of 32 years. In the only previously reported case of symptomatic tauopathy in a monkey, gait disturbance, trembling, drowsiness, and reduced activity were observed after at least 35 years of age (Kiatipattanasakul et al., 2000). The human age equivalent of a 32-year-old macaque remains uncertain. However, given that female monkeys undergo menopause at approximately 25 years of age and that the maximum documented lifespan in captivity is approximately 40 years, it is reasonable to consider the 30s as an advanced age in macaques (Mattison and Vaughan, 2017). These findings raise the possibility that the parkinsonian features observed in the present case may be associated with age-related tau pathology.

A series of postmortem examinations in aged monkeys has revealed that tau pathology initially appears in the medial temporal lobe around the age of 20 years, and is accompanied by amyloid plaques (Oikawa et al., 2010). By contrast, older monkeys (over 30 years of age) exhibit a different distribution pattern of tau pathology that predominantly involves the basal ganglia and frontal regions (Uchihara et al., 2016). Notably, in monkeys presenting with parkinsonism, tau pathology is predominantly observed in the brainstem, diencephalon, and basal ganglia (Kiatipattanasakul et al., 2000). This distribution pattern has been interpreted as more closely resembling that of PSP rather than that of AD. In our case, tau pathology was also distributed from the brainstem to the diencephalon, with prominent involvement of the tegmental regions of the midbrain and pons as well as the reticular formation of the medulla oblongata. Consistent with previous reports, confocal imaging demonstrated not only neuronal inclusions but also abundant glial inclusions (Kiatipattanasakul et al., 2000). The predominance of 4-repeat tau and the presence of astrocytic tau pathology further support the idea that this case exhibited a PSP-like pattern of tau pathology, although the morphology of the astrocytic inclusions differed from the classic tufted astrocytes that are typically observed in PSP.

Accumulation of 4-repeat tau with a PSP-like pathological pattern has also been reported in aged cynomolgus monkeys without neurological symptoms, based on immunohistochemical and biochemical analyses (Uchihara et al., 2016). Consistent with these observations, PSP-like tau pathology has also been identified in elderly humans without a clinical diagnosis of PSP in large forensic autopsy series, demonstrating neuronal and glial 4-repeat tau inclusions in the basal ganglia and brainstem (Yoshida et al., 2017). Together, these findings indicate that PSP-like tau pathology can emerge as an age-associated process and may remain clinically silent for a prolonged period in many cases, rather than reflecting an increased intrinsic susceptibility of macaques to PSP-like tauopathy. On the other hand, regarding tau expression, changes in the ratio of 3R/4R isoforms have also been reported in human sporadic 4R tauopathy (Ingelsson et al., 2007). In the case of Monkey T, SNVs in the MAPT gene UTR and altered mRNA expression may have contributed to the accumulation of 4R tau, however, further investigation is required to elucidate the underlying mechanisms.

Although parkinsonian features emerge in PSP seed-injected macaque models that recapitulate PSP-like pathology, only a mild loss of dopaminergic neurons (a 19.3% reduction) is observed in the substantia nigra (Darricau et al., 2023). These findings suggest that glial tau pathology can contribute directly to motor symptoms even in the absence of overt neuronal loss. Similarly, in the present case, Monkey T exhibited depigmentation of the substantia nigra compared with Monkey A; however, there was no marked loss of TH-positive neurons, as is typically observed in the brains of patients with parkinsonian disorders. In addition, no overt loss of TH-positive fibers was detected in the striatum. By contrast, abundant tau pathology was observed in the brainstem, which may have contributed to the development of parkinsonian features. The lack of overt dopaminergic neuronal loss is also in line with the absence of prominent reactive astrogliosis. As reactive astrogliosis varies with the severity and temporal dynamics of central nervous system injury (Sofroniew and Vinters, 2010), the slowly progressive nature and limited extent of neurodegeneration in this case may have resulted in an attenuated astroglial response.

The present study has several limitations. First, behavioral assessments were limited to video-based observations because invasive recordings were not feasible; Both monkeys used in the present study had previously participated in the same, separate study. Cognitive function was unable to be evaluated, which is a notable limitation given its relevance to human tauopathies. Additionally, this study is based on a single pathological case, which reflects the rarity of symptomatic tauopathy in macaques. Although it remains difficult to determine what distinguishes this symptomatic case from previously reported asymptomatic macaques with tau pathology, the advanced age of the animal may have allowed widespread tau pathology to progress to neurodegeneration and brain atrophy, ultimately leading to the onset of clinical symptoms. Another limitation is the lack of neuroimaging data because our facility is not equipped to perform such scans in non-human primates. Although we confirmed the absence of pathogenic *MAPT* mutations, the scope of our genetic analysis was also limited, highlighting a broader limitation in macaque research caused by insufficient genomic resources.

The present study is important in that it presents a rare pathological analysis of an aged macaque—a condition that is extremely difficult to reproduce. This case may represent an intermediate phenotype between age-associated asymptomatic tau deposition and experimental seed-induced models, thus further supporting the relevance of macaques in tauopathy research. Our findings strengthen the rationale for using macaque monkeys as a model for tauopathy research.

## Data Availability

The raw data supporting the conclusions of this article will be made available by the authors, without undue reservation.

## Footnotes

1
http://fiji.sc

2
https://ensembl.org/

## Supplementary material

SUPPLEMENTARY FIGURE 1 Western blot analysis of phosphorylated tau in the two monkeys. Western blot analysis of sarkosyl-insoluble tau fractions prepared from the pons of Monkey T and Monkey A using an identical protocol. Immunoblotting was performed using the AT8 antibody to detect phosphorylated tau. Equal amounts of tissue were used for each preparation.

SUPPLEMENTARY VIDEOS 1, 2 Tremor in the extremities in Monkey T. Resting tremor in the extremities of Monkey T.

SUPPLEMENTARY VIDEO 3 Monkey T exhibiting a flexed posture. Before L-DOPA administration, Monkey T was unable to adopt a standing posture.

SUPPLEMENTARY VIDEO 4 Monkeys A and T. Monkey A was observed to be ambulatory, whereas Monkey T remained in a flexed posture with limited movement.

SUPPLEMENTARY VIDEO 5 Behavior of Monkey A. Monkey A did not exhibit neurological symptoms.

SUPPLEMENTARY VIDEO 6 Effects of L-DOPA on the flexed posture. After L-DOPA administration, Monkey T was able to adopt a standing posture.
