# Supplementary figures and images for: Spontaneous tauopathy with parkinsonism in an aged cynomolgus macaque
Source: Front Aging Neurosci. 2026 Jan 28;18:1715911. doi: 10.3389/fnagi.2026.1715911 (PMC12895427; doi:10.3389/fnagi.2026.1715911)

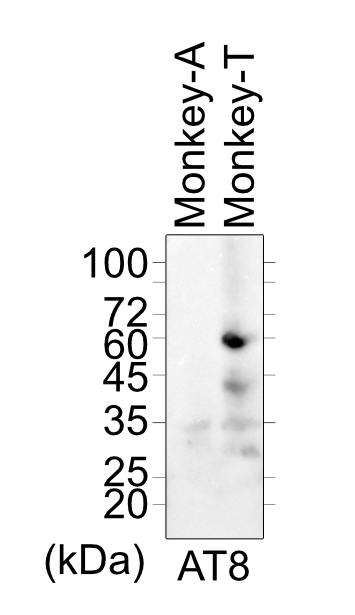

Supplement: SUPPLEMENTARY FIGURE 1 — Western blot analysis of phosphorylated tau in the two monkeys. Western blot analysis of sarkosyl-insoluble tau fractions prepared from the pons of Monkey T and Monkey A using an identical protocol. Immunoblotting was performed using the AT8 antibody to detect phosphorylated tau. Equal amounts of tissue were used for each preparation. [file Image_1.TIFF]
