# Supplementary material for: Spontaneous tauopathy with parkinsonism in an aged cynomolgus macaque
Source: Front Aging Neurosci. 2026 Jan 28;18:1715911. doi: 10.3389/fnagi.2026.1715911 (PMC12895427; doi:10.3389/fnagi.2026.1715911)
Supplement: Supplementary file 8 [file Table_1.DOCX]

Supplementary Material

# Supplementary Table

**Table 1. Primer sequences for *MAPT* sequencing analysis**

| Primer name | Sequence |
| --- | --- |
| macaque_MAPT_exon01_F | GGCCTGAGGCGTAGGGG |
| macaque_MAPT_exon01_R* | CAGTCTGTGGAGGCTGAGGG |
| macaque_MAPT_exon02_F* | CTGCCATGAACTGGGAGGAG |
| macaque_MAPT_exon02_R* | TGGATGCAAATTGTTCCTGC |
| macaque_MAPT_exon03_F* | TCCACAAGACACTGCTCCCC |
| macaque_MAPT_exon03_R | AGGAGGGAGGCTCAAAGCAC |
| macaque_MAPT_exon04_F* | CACTCCTCCTCCCTGCATTG |
| macaque_MAPT_exon04_R* | CACTTCTGTCGCAGGTCAGC |
| macaque_MAPT_exon05_F | CCAGAAGACACAGAGAGCTTGG |
| macaque_MAPT_exon05_R* | CTTTTCAGGGCAGAGCCCAC |
| macaque_MAPT_exon06_1_F* | GACGAAAAGGTGGGGACTGG |
| macaque_MAPT_exon06_1_R* | GAGGAAATCCACAGGGAGGG |
| macaque_MAPT_exon06_2_F* | CAGGGTCTCCCCAGTCCAAG |
| macaque_MAPT_exon06_2_R* | GGAGTTGGGAAGGCCTGAAG |
| macaque_MAPT_exon07_F* | TCACCACAGCTGGCTGTTTC |
| macaque_MAPT_exon07_R | TGAGAATGAATCAGACATAAAGCACAG |
| macaque_MAPT_exon08_F* | TGCTTTCAACCATTACCTGCC |
| macaque_MAPT_exon08_R | CATTTGAAGAAAGGTCTTTGAGGC |
| macaque_MAPT_exon09_F* | GCTGGTGTTGACTCGGTGG |
| macaque_MAPT_exon09_R | AGGGGCCGTTCACTCTCAG |
| macaque_MAPT_exon10_F* | GCCTGCCATGTGAAGGACTC |
| macaque_MAPT_exon10_R | AGGGTCCTGTCGGCTCTAGG |
| macaque_MAPT_exon11_F* | GAGCCTGGGAATGGACCTG |
| macaque_MAPT_exon11_R | CTACCCTTCCAGGCGCAG |
| macaque_MAPT_exon12_F | AAAAGTGGAGGCATCCTTGC |
| macaque_MAPT_exon12_R* | CAAGTGTACGCACTCACGCC |
| macaque_MAPT_exon13_F* | TCCCATTTTATCCTCTTTGTCTCTC |
| macaque_MAPT_exon13_R | TTCGAAGTCTGGGGCAGTTC |
| macaque_MAPT_exon14_F* | TGCCCTGTAGACCACAGACCTC |
| macaque_MAPT_exon14_R | GGACCAGTCTTTGTCCACTCG |
| macaque_MAPT_exon15_1_F* | CAGTTGGCAGGGCTGGTC |
| macaque_MAPT_exon15_1_R* | CTGTGGCTCCACGAACACAC |
| macaque_MAPT_exon15_1_F’* | CCAAGCAGGGTTTGTGATC |
| macaque_MAPT_exon15_1_R’* | TGATGAACCGATTAACCGAACT |
| macaque_MAPT_exon15_2_F* | AGGCTGTGAAAGCTGCTTCG |
| macaque_MAPT_exon15_2_R* | ACACTCCAGAGATGCCGGTG |
| macaque_MAPT_exon15_3_F* | CCACTTTGCAGACCTGGGAC |
| macaque_MAPT_exon15_3_R* | GGGCTGATTCTATGCATGTGG |
| macaque_MAPT_exon15_4_F* | AGGGCAGGCCCACAGTC |
| macaque_MAPT_exon15_4_R* | TGGCACTGGCTTCCTTCTTC |
| macaque_MAPT_exon15_5_F* | CTTGTGGCTGGTCTGGCTTG |
| macaque_MAPT_exon15_5_R* | AATGCAGCCGAAACTGTTGG |
| macaque_MAPT_exon15_6_F* | GCTGTTCGCTAAGTCCCAGC |
| macaque_MAPT_exon15_6_R* | ATTGCTACGCCTGCACCTG |
| macaque_MAPT_exon15_7_F* | AGCAGCCACGCGGTTAGAG |
| macaque_MAPT_exon15_7_R* | TTCTCTAAGGGTGAGTGGGAAAG |

The primer names labeled with an asterisk indicate the primers used in Sanger sequencing.

**
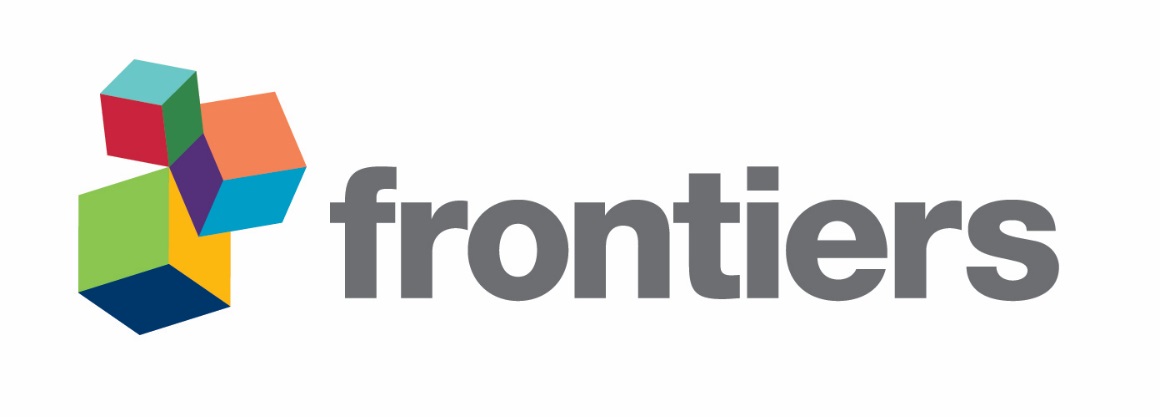
**
